# Attentional Load Modulates Responses of Human Primary Visual Cortex to Invisible Stimuli

**DOI:** 10.1016/j.cub.2007.01.070

**Published:** 2007-03-20

**Authors:** Bahador Bahrami, Nilli Lavie, Geraint Rees

**Affiliations:** 1 Institute of Cognitive Neuroscience, University College London, Alexandra House, 17 Queen Square, London WC1N 3AR, United Kingdom; 2 Department of Psychology, University College London, 26 Bedford Way, London WC1H 0AP, United Kingdom; 3 Wellcome Department of Imaging Neuroscience, Institute of Neurology, University College London, 12 Queen Square, London WC1N 3BG, United Kingdom

**Keywords:** SYSNEURO

## Abstract

Visual neuroscience has long sought to determine the extent to which stimulus-evoked activity in visual cortex depends on attention and awareness. Some influential theories of consciousness maintain that the allocation of attention is restricted to conscious representations [1, 2]. However, in the load theory of attention [3], competition between task-relevant and task-irrelevant stimuli for limited-capacity attention does not depend on conscious perception of the irrelevant stimuli. The critical test is whether the level of attentional load in a relevant task would determine unconscious neural processing of invisible stimuli. Human participants were scanned with high-field fMRI while they performed a foveal task of low or high attentional load. Irrelevant, invisible monocular stimuli were simultaneously presented peripherally and were continuously suppressed by a flashing mask in the other eye [4]. Attentional load in the foveal task strongly modulated retinotopic activity evoked in primary visual cortex (V1) by the invisible stimuli. Contrary to traditional views [1, 2, 5, 6], we found that availability of attentional capacity determines neural representations related to unconscious processing of continuously suppressed stimuli in human primary visual cortex. Spillover of attention to cortical representations of invisible stimuli (under low load) cannot be a sufficient condition for their awareness.

## Results and Discussion

**Figure 1:**
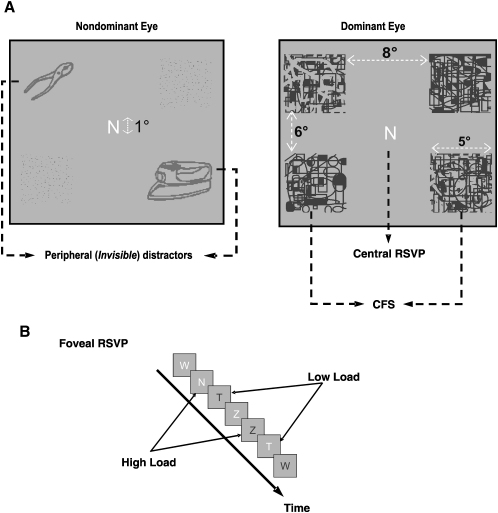
Stimulus Configuration Used in the Experiment (A) A continuous central RSVP task was combined with presentation of invisible, task-irrelevant stimuli in the periphery. While a subject wore red-blue anaglyph glasses, the nondominant eye was presented with low-contrast red line drawings of two objects in two of the four visual-field quadrants along the 45° or 135° diagonal. The dominant eye was presented with four highly salient, high-contrast, and rapidly changing blue masks, one in each of the four quadrants. (B) Rapid serial visual presentation (RSVP) task at the fovea. Depending on task instruction, the same pseudorandom stream of colored letters served as both the low-load (detection of “pop out” target letter ‘T’ irrespective of its color) and high-load (detection of specific “conjunctions” of letter form and color, i.e., white N and blue Z) tasks.

Participants viewed a central letter stream presented binocularly at fixation and performed tasks of either low (monitoring for a single *pop-out* feature) or high (monitoring for “conjunctions” of letter shape and color features) attentional load in separate 20 s blocks (see Experimental Procedures and Figure 1B). During task performance, low-contrast tool images were presented continuously to one eye in two of the four visual quadrants (either on the 45° or 135° diagonal throughout a block) while high-contrast, rapidly changing masks were presented to the other eye in all four quadrants (Figure 1A). This configuration produces prolonged continuous flash suppression (CFS) and resultant invisibility of the monocular images [4, 7]. The strength of the tool images was chosen per participant for both load conditions on the basis of a prescanning session that established the stimulus contrast that generated chance performance in the low-load condition (see Experimental Procedures).

**Figure 2:**
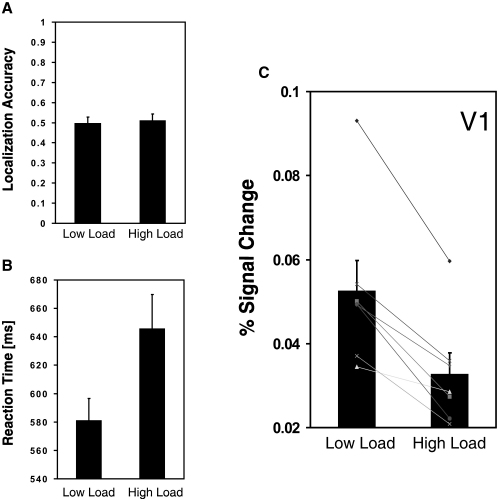
Experimental Results (A) In both load conditions, localization of the tool images occurred at the level of chance (50%; one-sample t test; p = 0.919 and 0.737 for low and high loads, respectively). Individual analyses also confirmed that none of the participants were better than chance. (B) Reaction time for RSVP target detection as a function of load. Observers were significantly slower under high-load conditions (paired t test; *t*(6) = 5.792; p = 0.001). (C) Differential V1 BOLD response to invisible images under high and low loads. The *y* axis shows the percent signal change, averaged over the selected V1 voxels, for suppressed-stimulus presence minus absence. Data are plotted for each participant (lines) as well as the group means (bars). All error bars indicate 1 standard error of the mean.

We established the invisibility of the tool stimuli during scanning by requiring participants to make two-alternative forced choices as to which diagonal had the tool images at the end of each 20 s block. Performance was consistently indistinguishable from the chance level in both load conditions (Figure 2A). Note that here we use a strict criterion for establishing invisibility. Because the tool images were displayed in the same locations throughout each block, correct detection could be made even if participants momentarily detected a fragment of the hidden objects.

Load manipulation at fixation was effective; reaction times were significantly longer in the high- (versus low-) load conditions (Figure 2B). We proceeded to analyze the fMRI data to determine whether load manipulation affected processing of the task-irrelevant invisible tool stimuli. First, we identified voxels corresponding to the retinotopic location of the quadrants in early visual cortex (V1, V2, and V3) by using a quadrant localizer (see Experimental Procedures; see also Figure S1 in the Supplemental Data available online). Then for each quadrant in each participant, we selected the subset of voxels whose activity reflected the retinotopic representation of the invisible tool images by comparing those blocks in which an invisible stimulus (plus CFS mask) was present in a particular quadrant with those in which only the CFS mask was present (in which case the invisible stimuli were on the opposite diagonal; see Experimental Procedures). Note that in this way the voxel-selection contrast is independent of and orthogonal to the load conditions.

Responses to the presence (versus absence) of the invisible objects in retinotopic V1 were significantly reduced under high load in the central task (Figure 2C; one-tailed paired t test, *t*(6) = 5.834; p < 0.001). The same analyses for V2 and V3 showed similar trends, but these did not reach statistical significance (for V2, *t*(6) = 1.319 and p = 0.12; for V3, *t*(6) = 1.774 and p = 0.063; one-tailed paired t test; see Figure S2). These findings clearly demonstrate that even subliminal retinotopic activity evoked by invisible stimuli in human primary visual cortex depends on attentional capacity.

Because the invisible stimuli were presented in two of the four quadrants and were clearly separated from the foveal representation of the fixated letters in the attentional task, we could ascertain that the attentional modulations found were specific to representations of the suppressed images (rather than foveal representations of the central task stimuli). Moreover, because we have shown that attentional load modulates V1 response associated with the presence (versus absence) of the invisible stimulus (namely, an interaction between load and V1 response to the invisible stimulus), the results clearly show specific effects of load on the neural processing of the invisible stimuli.

Furthermore, within the same voxels that responded to the presence of invisible stimuli, BOLD responses elicited by the CFS mask alone (i.e., in the quadrants with no suppressed stimulus) were not significantly affected by load (p > 0.40 for V1, V2, and V3; see Figure S3). This provides further confirmation that the load modulation observed was specific to the neural representations of the suppressed stimuli.

Thus, our new findings clearly demonstrate that the effects of attentional load are not confined to neural representations that have reached conscious awareness and extend load theory to the case of neural V1 processing that does not invoke conscious perception. Previous behavioral results are inconclusive with regard to the role of attentional load in unconscious perception. Unconscious priming effects can be eliminated with tasks of high attentional load [8, 9] as long as the prime stimulus is uncued [9]. However, because priming effects are measured via the influence of certain stimulus-response associations on reaction times, these effects may reflect the impact of attention on the strength of motor-response associations for unconscious stimuli (see [10–12] for recent evidence for this account). As such, these studies remain inconclusive with regard to the question of whether attentional load determines early unconscious perceptual representations such as those mediated by retinotopic V1 activity (as here).

Previous functional-imaging studies have shown that high perceptual load in a task modulates activity related to task-irrelevant stimuli in primary visual cortex [13, 14]. However, it is controversial whether V1 activity reflects unconscious [15, 16] or conscious perception [17–19]. Indeed, some have even claimed that V1 activity related to feedback from extrastriate cortices serves as the arbiter of conscious awareness [20]. The present findings are the first to show that neural processes involved in the retinotopic registration of stimulus presence in V1 depend on availability of attentional capacity, even when they do not invoke any conscious experience. These findings challenge previous suggestions that attention and awareness are one and the same [5, 6] or that attention acts as the gate-keeper to awareness [1, 2]. Importantly, our new findings that the level of attentional load in a central task determines retinotopic V1 responses to invisible stimuli clarify both that unconscious processing depends on attentional capacity (which is reduced in conditions of high load) and that availability of attentional capacity for stimulus processing (in the low load conditions) cannot be a sufficient condition for awareness.

## Experimental Procedures

### Participants

Seven healthy volunteers (four female; mean age of 26.5 years; range of 22–34 years) gave written informed consent to participate in the experiment, which was approved by the local ethics committee. All participants were naïve to the purpose of the experiment and had normal color vision and normal or corrected-to-normal visual acuity.

### Foveal Attentional Load Task

A continuous stream of rapid serial visual presentation (RSVP) of letters colored blue or white was displayed at fixation (Figures 1A and 1B). Participants monitored for targets (low-load condition, “T” of either color; high-load condition, blue “Z” and white “N”—but not the opposite conjunctions of color and letter shape) within a stream of 20 successive letters randomly chosen from T, N, Z, M, L, K, and W and randomly colored blue or white (Arial, font size = 25; visual angle: 1°) and responded by pressing a button. Each letter was presented for 250 ms followed by a 750 ms blank period. Based on previous work [14, 21, 22], we reasoned that detecting the target letter ‘T’ (irrespective of its color) involves detecting a single “pop-out” form feature and requires low levels of attentional demand, whereas monitoring for letters of similar form (N and Z) while also looking for a specific color for each imposes higher levels of attentional load. This allowed us to use the exact same pseudorandom stream of central stimuli (reshuffled for each run) for both high and low loads (Figure 1B). Target items in one condition appeared as distracters in the other condition with the same frequency. Within each block, 2–4 targets were embedded in the sequence so that the number of detected targets would not lead to predictability.

### Invisible Images and Continuous-Flash Suppression

For each block, two line drawings of man-made tools were randomly chosen from a subset of the Snodgrass-Vanderwart set [23]. When invisible, objects in this category can evoke measurable fMRI activation in the human dorsal visual stream [7]. Throughout each block, two such images were displayed in two diagonally opposite quadrants (Figure 1A, left). Blue CFS stimuli were then superimposed on the four quadrants. CFS stimuli consisted of maximum-contrast, rapidly flashing (10 Hz), randomly placed geometrical shapes; contours and moving dots alternated with occasional uniform blue patches covering the whole quadrant. Participants viewed these composite images with red-blue anaglyph eyeglasses such that the red images and the blue CFS stimuli were exclusively presented to the nondominant and dominant eye, respectively. To enhance sensitivity to invisible objects, we added an additional feature to the CFS: within each block, the contrast of the invisible objects fluctuated (with a frequency of approximately 0.2 Hz) between zero and the participant-specific, subthreshold maximum (see “Procedure” section) so that adaptation was avoided. This provided frequent (yet still invisible) *transient onsets* of the suppressed image. Stimuli with multiple onsets drive the BOLD signal more strongly than steady stimuli. All visual stimuli were back projected by an LCD projector (NEC LT158) on a screen that was viewed in a mirror mounted on the MRI head coil (total display size 26° × 22° of visual angle, 1024 × 768 pixel screen resolution, 60 Hz refresh rate, background luminance = 11 Cd/m^2^). Stimuli were generated with the Cogent Toolbox (Cogent, www.vislab.ucl.ac.uk/Cogent/) for MATLAB (Mathworks).

### Procedure

Each block started with a blank screen and a fixation point displayed in the center for 500 ms. The central RSVP and the peripheral CFS then started simultaneously and were presented for 20 s. During the block, participants performed the RSVP task. At the end of the block, a brief (2000 ms) display in which the quadrants along 45° and 135° diagonal were labeled 1 and 2, respectively, appeared. Subjects reported which diagonal they thought most likely contained the invisible images by pressing one of two keys. The actual location of the invisible objects was randomized across blocks.

Each experimental run started with task instructions followed by eight blocks of the same load type; new instructions were then displayed, and each participant completed another eight blocks, altogether lasting 572 s. Consecutive blocks were separated by a 15 s blank display with a fixation point in the center. Each participant completed four to six runs in a counterbalanced order.

Prior to scanning, each observer participated in a short (15 trials for each load condition) staircase threshold-estimation procedure outside the scanner. Using an accelerated stochastic approximation method [24], we obtained a rough estimate of suppressed stimulus Michelson contrast that enabled around 75% correct localization. Once inside the scanner, each participant completed 3–5 practice runs (approximately 30 min altogether) starting with maximum-contrast, clearly visible red images embedded in the CFS so that the subject became familiarized with the scanner conditions. Then contrast was reduced to 67% of the previously estimated threshold under low load. Performance accuracy was monitored at the end of each practice run, and contrast was modified to reach the maximum contrast for which the participant still performed no better than chance in the 2-AFC localization task. It is worth noting that, except for the practice run, participants' subjective reports remained completely unaffected by these modifications, and they reported no awareness of hidden objects after debriefing.

### fMRI Acquisition and Analysis

Data were acquired with a 3T Allegra MRI scanner (Siemens Medical Systems). For the main experiment, four to six runs of 275 volumes were collected per participant (32 axial slices; TR = 2.08 s; resolution 3 × 3 × 3 mm). We acquired a T1-weighted volume to allow coregistration of functional data with the individual participants' structural scans. In a second session, we collected two runs of 165 volumes for the same participants for retinotopic mapping and two runs of 160 volumes for quadrant localization. During the retinotopic-mapping runs, participants viewed standard checkerboard stimuli that covered the horizontal and vertical meridians.

We analyzed fMRI data by using SPM2 (http://www.fil.ion.ucl.ac.uk/spm). We discarded the first five images of each run to allow for magnetic-saturation effects. The remaining images were realigned, resliced, coregistered to the individual participants' structural scans, and spatially smoothed with a narrow Gaussian kernel of 5 mm full-width half-maximum (we smoothed only for the main experiment data). The data were high-pass filtered (the cut-off frequency was 0.0083 Hz) so that low-frequency signal drifts would be removed, and data were then submitted to a within-participant analysis with a voxel-wise general linear model (GLM) that comprised four delayed boxcar waveforms for each scanning run so that the mean activity evoked by each of the four combinations of experimental conditions as well as the motion correction parameters (as effects of no interest) could be extracted.

Regions of interest corresponding to early visual cortex (V1, V2, and V3) were identified for each participant (Figure S1, top panel) according to conventional meridian mapping methods [25–27]; Fourier analyses in SPM2 and segmentation and cortical flattening in mrGray (http://white.stanford.edu/∼brian/mri/segmentUnfold.htm) were used. In order to localize the retinotopic locations corresponding to our CFS stimuli (with or without invisible tool images presented in the other eye), we used a two-stage procedure. First, we employed an independent quadrant localizer. Participants maintained fixation while checkerboard patterns were displayed in two of the four quadrants (Figure S1, middle right and bottom right panels). Black and white squares within each checkerboard pattern alternated at a frequency of 10 Hz. In each stimulated quadrant, the pattern covered the same area as did the blue CFS stimulus in the main experiment (Figure 1A). Patterns were displayed either along the 45° (Figure S1, middle right) or the 135° (Figure S1, bottom right) diagonal for blocks of 20 s followed by 20 s of no stimulation. Each scanning run of 160 volumes consisted of eight blocks (four for each diagonal), and two runs were collected for each participant. These data were used for localizing peripheral voxels within each region of interest. For example, voxels corresponding to the topographic representation of the lower left quadrant (these were clustered in dorsal right V1, V2, and V3) were most responsive to stimuli aligned to 45° diagonal (Figure S1, middle panel). Conversely, within the same hemisphere, there were clusters in ventral V1, V2, and V3 that were most responsive to the opposite stimulus geometry (Figure S1, bottom panel). By superimposing statistical parametric maps extracted from the quadrant localizer on top of regions of interest (V1, V2, and V3) identified by meridian mapping and selecting the regions of overlap (schematically circumscribed in Figure S1 by dotted ellipses), we identified subregions corresponding to each quadrant. Having thus identified the retinotopic regions corresponding to the visual-field locations where the CFS stimuli (with or without invisible monocular tool images) were presented, we then further selected the subset of voxels that responded to invisible tool images. We achieved this by finding, for each contrast, those voxels for which activity (assessed via the GLM procedure described above) was greater in blocks where an invisible tool stimulus was present in that quadrant than in those blocks where it was not present (and where the tool stimuli were present instead on the opposite diagonal). We selected either the peak voxel for this contrast or the upper twentieth percentile of the most active voxels (yielding, on average, n = 4.5, 5, and 4.0 voxels/quadrant for V1, V2, and V3, respectively); results were qualitatively the same (and statistically significant) for either selection criterion, so for the results presented here, the latter criterion is used. Note that the voxel selection process described here is independent of the contrast of interest between high and low attentional loads.

## Supplemental Data

Document S1. Three Supplemental Figures
