# Supplementary material for: Attentional Load Modulates Responses of Human Primary Visual Cortex to Invisible Stimuli
Source: Curr Biol. 2007 Mar 20;17(6):509–13. doi: 10.1016/j.cub.2007.01.070 (PMC1885953; doi:10.1016/j.cub.2007.01.070)
Supplement: Document S1. Three Supplemental Figures [file mmc1.pdf]

Supplemental Data

Attentional Load Modulates Responses of Human Primary Visual Cortex to Invisible Stimuli

Bahador Bahrami, Nilli Lavie, and Geraint Rees

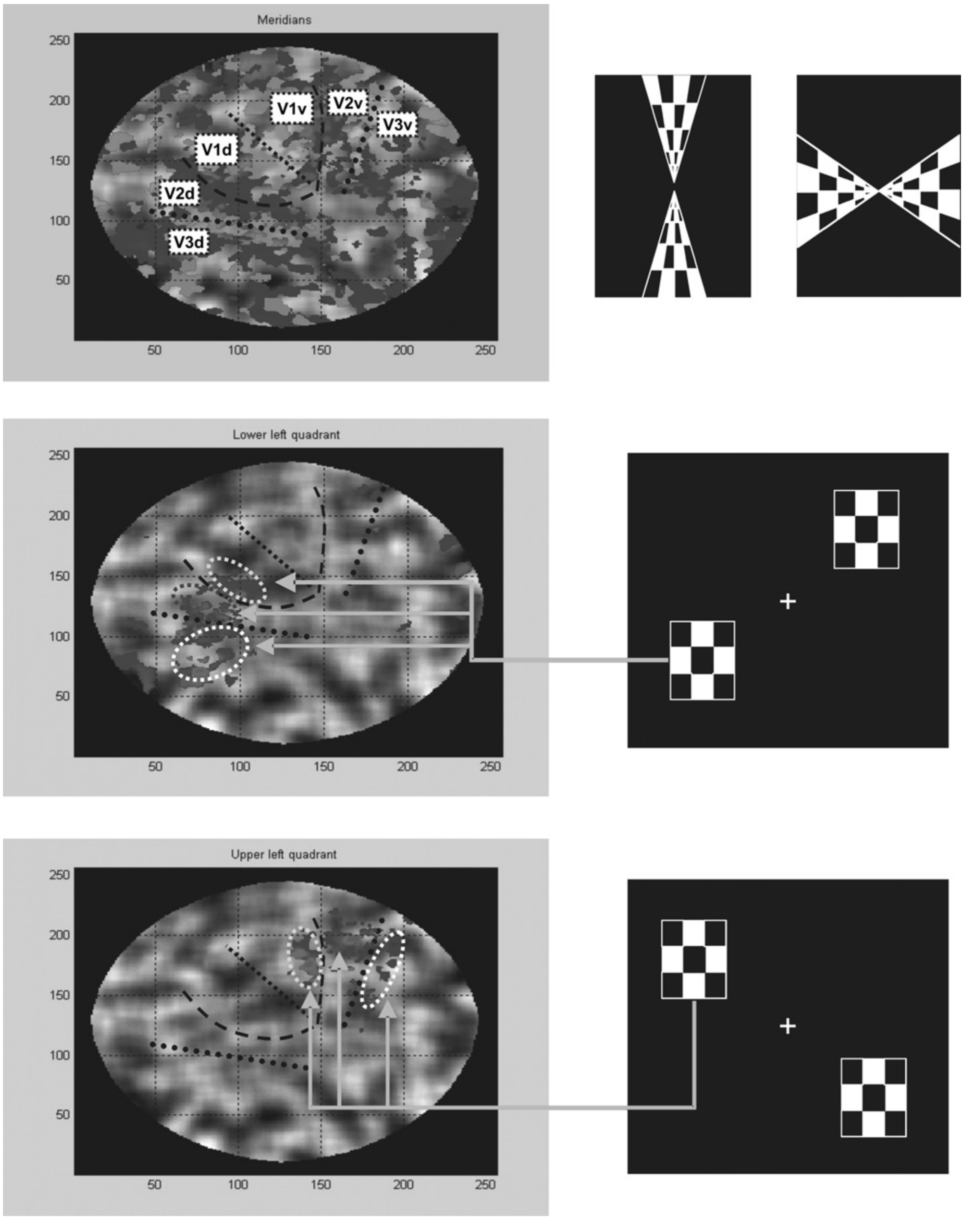

Figure S1. Quadrant Localization

Two-stage procedure used for localization of retinotopic representation of quadrants in V1–V3 in the flattened right occipital cortex of a representative subject. Top panel: meridian mapping. Middle and lower panels: checkerboards flashing in the lower and upper left hemifield activate the dorsal and ventral parts, respectively, of right V1, V2, and V3.

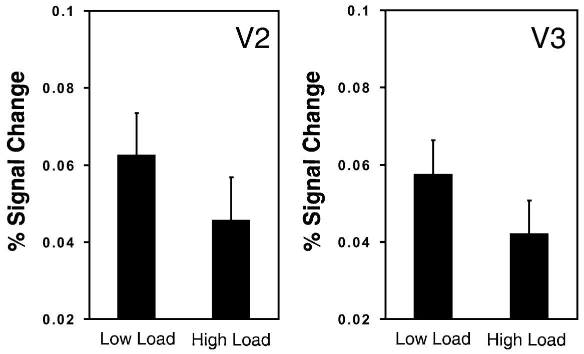

Figure S2. Differential BOLD Response in V2 and V3 to Invisible Images under High and Low Load

V2 is on the left, and V3 is on the right. The *y* axis shows the percent signal change, averaged over the selected voxels, for suppressed-stimulus presence minus absence. All error bars indicate 1 standard error of the mean.

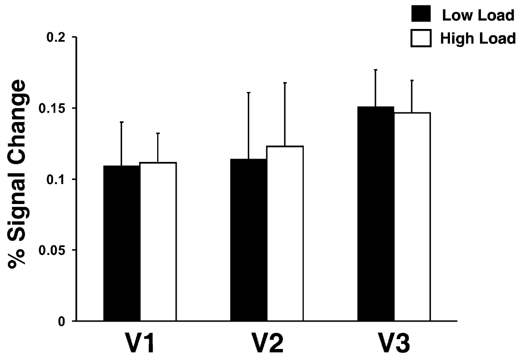

Figure S3. Response of the Visual Cortex to CFS

BOLD response to CFS stimulus in the *empty* (i.e., no suppressed stimulus) quadrants did not show any significant difference between high and low load in any of the regions. For V1, $t(6) = 0.151$ and $p = 0.80$; for V2, $t(6) = 0.885$ and $p = 0.41$; for V3, $t(6) = -0.314$ and $p = 0.70$; paired *t*-test. All error bars represent standard error of the mean.
